# Comparison of the Moist Material Relative Permittivity Readouts Using the Non-Invasive Reflectometric Sensors and Microwave Antenna

**DOI:** 10.3390/s22103622

**Published:** 2022-05-10

**Authors:** Zbigniew Suchorab, Krzysztof Tabiś, Przemysław Brzyski, Zenon Szczepaniak, Tomasz Rogala, Waldemar Susek, Grzegorz Łagód

**Affiliations:** 1 Faculty of Environmental Engineering, Lublin University of Technology, Nadbystrzycka 40B Str., 20-618 Lublin, Poland; z.suchorab@pollub.pl (Z.S.); g.lagod@pollub.pl (G.Ł.); 2 Aquapol Polska CPV, Żeromskiego 12 Str., 58-160 Świebodzice, Poland; ktabis@aquapol.pl; 3 Faculty of Civil Engineering and Architecture, Lublin University of Technology, Nadbystrzycka 40 Str., 20-618 Lublin, Poland; p.brzyski@pollub.pl; 4 Faculty of Electronics, Military University of Technology, Gen. Sylwestra Kaliskiego 2 Str., 00-908 Warsaw, Poland; zenon.szczepaniak@wat.edu.pl (Z.S.); waldemar.susek@wat.edu.pl (W.S.)

**Keywords:** time domain reflectometry, microwaves, microwave antenna, relative permittivity, moisture detection

## Abstract

The article concerns the issue of non-invasive moisture sensing in building materials. Two techniques that enable evaluating the value of the relative permittivity of the material, being the measure of porous material moisture, have been utilized for the research. The first is the microwave technique that utilizes the non-contact measurement of velocity of microwave radiation across the tested material and the second is the time domain reflectometry (TDR) technique based on the measurement of electromagnetic pulse propagation time along the waveguides, being the elements of sensor design. The tested building material involved samples of red ceramic brick that differed in moisture, ranging between 0% and 14% moisture by weight. The main goal of the research was to present the measuring potential of both techniques for moisture evaluation as well as emphasize the advantages and disadvantages of each method. Within the research, it was stated that both methods provide similar measuring potential, with a slight advantage in favor of a microwave non-contact sensor over surface TDR sensor designs.

## 1. Introduction

### 1.1. Problem of Moisture Presence in the Building Envelopes

Water presence in building envelopes is a typical phenomenon that can be a serious exploitation problem. It may occur in historical buildings, but also in newly built houses. The major sources of water presence are: inappropriate building construction, poor ventilation, as well as failures of water supply systems and flooding [1,2,3,4].

Water can appear inside the envelopes under several forms. It may be present as free water, chemically bound water, physically bound water, and water vapor, condensation and capillary water [5,6]. Free water is the factor which has the greatest influence on the performance of objects and most harmful for buildings. When contained inside the envelopes, it may enhance the value of material thermal conductivity coefficient [7,8], significantly influencing the energetic performance of the buildings [9]. The water present in building barriers can be also divided into condensation water and capillary water. The source of the condensation water is a lack or improper performance of the ventilation system. On the other hand, the presence of the capillary water mainly means the influence of ground water rise in the porous structure of the material and lack or destruction of the horizontal or vertical water-proof isolation [10,11,12,13,14,15].

The water that is present inside the building materials causes the construction decay in several processes that can be divided into the physical, chemical, and biological [16]. Among the physical-chemical processes, frosting and defrosting [17,18,19] should be mentioned, in addition to shrinking and expanding [20,21] as well as mechanical damage caused by salt crystallization [22,23,24]. Moreover, the phenomenon of metal corrosion caused by water which occurs mainly in the reinforcing steel. Finally, the destruction of the finishing materials must be mentioned among the physical-chemical processes [25,26].

Biological processes represent another reason for wearing out of building barriers due to the excessive water presence. The microorganisms present in moist materials cause the decay of the building envelopes with the most dangerous fungal development [1,27,28,29], which negatively influences the indoor air parameters. Currently, this problem becomes more important due to the increased amount of time (90%) spent by people indoors, where the pollutant concentrations are significantly higher compared to the outdoor air [30].

All the above-mentioned consequences of moisture presence in the buildings envelopes that negatively influence their performance, mainly the deteriorating indoor air quality, represent the cause of so-called sick building syndrome (SBS), often caused by fungal metabolites, predominantly microbial volatile organic compounds (mVOC), mycotoxins, spores, etc. [16,29,31]. Another problem is the mechanical degradation of the masonry, which in turn may be the reason for a building demolition.

### 1.2. Material Relative Permittivity

The structure of the porous building material consists of the three phases, namely solid, liquid, and gaseous [32,33]. Each phase has a certain value of relative permittivity. The relative permittivity of a three-phase mixture is the result of the relative permittivity of individual phases. The relative permittivity values of the individual phases of building structures are as follows: air, 1; solid phase, 1–15; water, 80 [34,35,36]. Concerning the building materials, the values of relative permittivity are the following: according to Davis and Annan [37], the relative permittivity of granite equals 4–6, clay—5–10 and sand—3–5. According to Daniels [38], the relative permittivity of granite equals 5, sandstone 2–3, clay 2–6, and sand 4–6. Korhonen et al. [39] evaluated the relative permittivity of sand as equal to 2.9, cement 2.7, dry concrete 5.4, and dry mortar 3.9. Finally, Mohamed [40] gives the value of relative permittivity of the following minerals: calcite 6.4–8.5, gypsum 6.5, orthoclase 5.6, and finally quartz 4.5. As can be noticed, the relative permittivity of water clearly differs from that of other media. This is a consequence of the asymmetric charge distribution in its molecule and its polar structure [41].

The dielectric permittivity ε is a measure of the behavior of matter particles after the application of an external, alternating electric field. Due to the application of an electric field to the medium, the water molecules rotate in the direction of the applied field. The consequence of this ordering of dipoles is the accumulation of energy released when the electric field disappears [34].

The electrical permittivity of the materials is a complex number that consists of a real (*ε*′) and an imaginary (*ε*″) part. The real part describes the baseline value of moisture estimation using dielectric techniques, namely the amount of energy released in the alternating field, and the imaginary part includes energy losses due to ionic conductivity, which are strongly dependent on material salinity [42]. The complex relative permittivity of a lossy medium can be calculated according to the following extended formula [32,41,43,44,45,46]:

(1) $$\varepsilon_{r}=\varepsilon_{r}^{\prime }-i\left(\varepsilon_{r}^{\prime \prime }+\frac{\sigma_{0}}{\varepsilon_{0}\omega }\right)$$

where *ε*′*_r_* denotes the real part of relative permittivity of medium, *ε*″*_r_* the imaginary part of relative permittivity of medium, *i* the imaginary unit (*i*^2^ = −1), *σ*_0_ the electrical conductivity [S/m], *ε*_0_ the permittivity of vacuum (*ε*_0_ = 8.85 × 10^−12^ F/m), and ω the angular frequency of the external electric field [rad/s].

The operating frequencies of microwave and reflectometric techniques utilized in the presented research reach the values between 1 and 8 GHz [44], which are high enough to decrease the influence of the imaginary part on the total value of the complex permittivity of examined medium, even if loaded with some ionic conductivity. Thus, it can be assumed that the ionic conductivity has low effect on the microwave as well as reflectometric moisture readouts. Salinity diminishes the pulse energy and the measuring markers are flatter. This feature can be also positively utilized for salinity evaluation of moist material, based on suitable signal attenuation interpretation [47].

### 1.3. Time Domain Reflectometry Technique for Evaluation of Moist Material Relative Permittivity Value

Time domain reflectometry (TDR) is an electric technique that utilizes the measurement of relative permittivity to evaluate the moisture of porous materials [33]. Measurement using this method consists in determining the travel time of the electromagnetic pulse along the rods of TDR probes inserted into the examined material. TDR probes usually consist of a coaxial cable, head, and measuring rods embedded in the tested material. The readings are based on marking the reflections on the individual discontinuities of the sensor waveguide, which are part of the sensor design. Typically, the described discontinuities are at the beginning and end of the probe. In detail, the performance of the TDR technique is described in the following articles [42,48,49,50].

Another approach to moisture evaluation, especially in rigid porous media such as building materials, is presented by surface, non-invasive sensors that are described in many studies [36,51,52,53,54,55,56]. Surface sensors are utilizable for moisture detection without the necessity of introducing the sensing elements inside the material. In comparison to traditional invasive probes, they provide worse accuracy and smaller sensitivity; on the other hand, they provide the readouts without changing the structure of the material.

The TDR method uses two types of pulses emitted by pulse generators: a stepped pulse [34] and a needle pulse [57]. The data collected by a TDR multimeter from the sensing probe consists in set of voltages distributed in time, called waveforms or reflectograms. In this research, a needle pulse generator with a rise time of 300 ps [57,58] that is more widely described in Section 2 and the exemplary waveforms achieved in the research are presented in Section 3. Waveform interpretation focused on the recognition of signal disturbances and propagation time enables to evaluate the relative permittivity of the materials according to the following formula [57]:

(2) $$\varepsilon_{r}=\left(\frac{c\cdot t_{p}}{2L}\right)$$

where *c* denotes the velocity of light in vacuum [m/s], *t_p_* the time of signal propagation [s], and *L* the distance between to marked points of the TDR sensor [m].

Establishing the value of the relative permittivity enables to evaluate the moisture of the material by applying theoretical or physical models available in literature [59,60,61] or individual calibration based on experimental tests [34,48,62,63,64].

### 1.4. Microwave Measurement Technique to Evaluate the Permittivity Value of the Moist Material

Application of microwave techniques for the determination of the permittivity of a material may be divided into three approaches. The first is the use of special measurement setup, mainly based on a microwave resonator. In this approach, a microwave resonating circuit, most frequently a cavity one, is loaded with a small sample of the investigated material [65]. The sample should be small enough or with low losses, in order to cause only a small disturbance of the electric field distribution inside a resonating cavity. This approach is called the field perturbation method. The resonator is connected as a two-port to the measurement setup, which most frequently is based on vector network analyzer (VNA). The measurement procedure relies on finding the transmittance versus frequency characteristics. Then, the modulus of S_21_ scattering parameter along the frequency carries the information about the resonance frequency value and the quality factor (Q-factor). There is a mathematical procedure to derive the values of sample permittivity from the measurements of resonant frequency shift and Q-factor values. However, this method requires physical contact with the material sample. Moreover, the sample must have a specific shape related to the resonator type (i.e., thin slab, cylinder, or powdered form). Non-destructive measurements in situ are not feasible.

The second common way is the use of a set of two antennas and performing the transmittance measurement through a sample or a full masonry wall. Then, the concept relies on the derivation of permittivity from the measurement results performed for the S_21_ scattering parameter. In this approach, there is an obvious need for accessing both sides of the measured object. Moreover, the calibration procedure is complicated here, and the method is sensitive to object thickness as well as the distance of antennas to the sample [66,67].

The third way is similar to the concept above but relies on the measurement of the reflection coefficient. In that case, only one antenna is needed, and the thickness of the sample may be different. The measurement of the complex value of reflectance S_11_ gives the information about material intrinsic impedance and complex permittivity [46].

**Figure 1:**
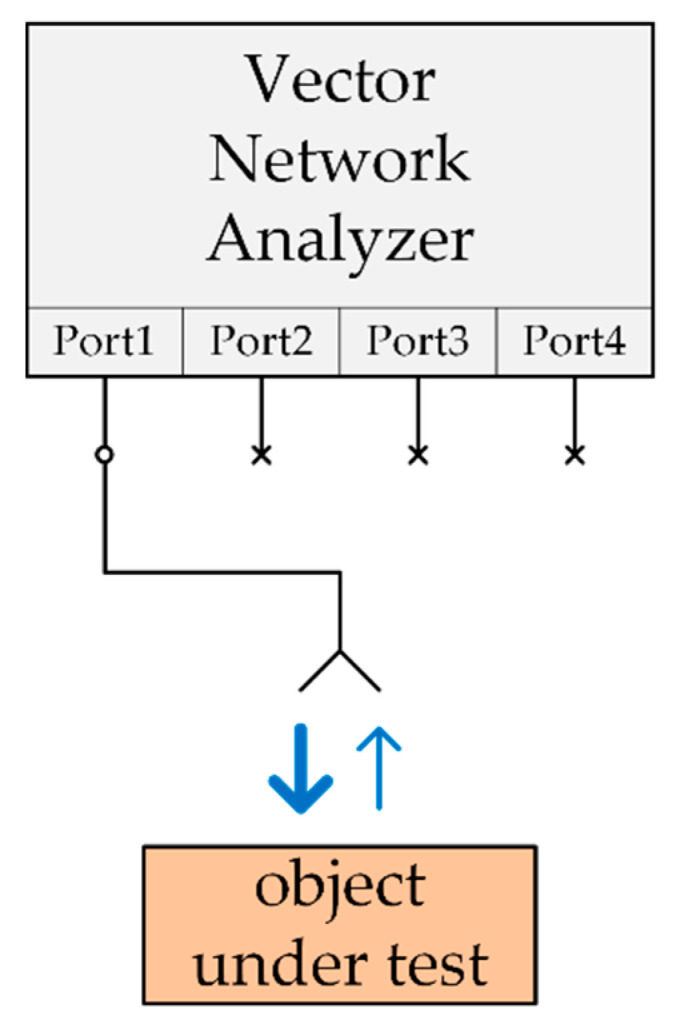
General diagram of microwave measurement technique based on reflection coefficient approach.

For the purposes of this work, the authors have chosen the concept of reflection coefficient measurement with the use of wideband antenna and VNA. The antenna is connected to only one port of the VNA (in the diagram connection marked with the symbol “◦” at port 1). After initial calibration the remaining ports of the VNA are not used during the tests (marked as “×” in the diagram). This approach allows measuring a building material sample with real thickness in a non-contact and non-destructive way. The diagram presenting the applied measurement technique is shown in Figure 1.

In this article, two sensing methods of non-invasive moisture detection have been compared for their efficiency to evaluate moisture in building materials. With the conducted research, the advantages and disadvantages of both reflectometric and microwave techniques have been revealed.

## 2. Materials and Methods

### 2.1. Samples Preparation

For the experiment, solid ceramic brick was used as a representative material widely employed for erecting the masonries. The bricks delivered from one production cycle have been used for the tests, in order to minimize technological influence on the results. The bricks subjected to the tests were characterized by a mass water absorption of approximately 14%_mass_. The aim was to prepare the samples with specific levels of absorbed water. This parameter was graded in 1% steps (15 bricks were prepared: each ranging from 0% to 14% of the gravimetric water content). In order to obtain specific gravimetric moisture status, the dry brick was evenly saturated with a suitable amount of water, controlling the mass using a laboratory scale, and then it was tightly wrapped in polyethylene foil so that the moisture has become evenly distributed over the entire volume of the brick. The seasoning period equaled 30 days. It was decided to prepare one sample for a given gravimetric water content, while five measurements were performed on each brick using both reflectometric and microwave methods. The mass moisture content w [%] is the mass of water absorbed by the material divided by the mass of dry material, in accordance with the formula [68]:

(3) $$w=\left(\frac{m_{wet}-m_{dry}}{m_{dry}}\right)\times 100\;[\%]$$

where *m_wet_* is the mass of brick in wet state [g] and *m_dry_* is the mass of brick in dry state [g].

Table 1 shows the brick apparent density and water dosage to obtain the assumed mass moisture content.

### 2.2. Time Domain Reflectometry Measuring Setup

**Figure 2:**
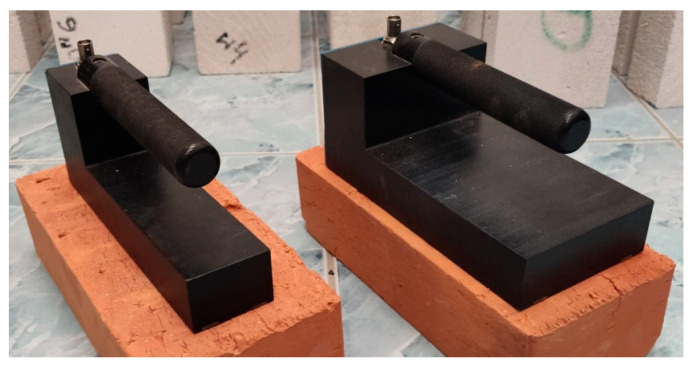
Photograph of non-invasive TDR sensors—Sensor A (**left**), Sensor B (**right**).

For the time domain reflectometry tests, the following elements were utilized: LOM TDR multimeter (ETest, Lublin, Poland), PC computer as controlling station and for data acquisition, as well as TDR surface sensors of own design connected with the TDR multimeter by a coaxial cable. Both applied TDR sensors are widely presented in authors’ article [56] and they differ in measuring waveguides spacing, where one (Sensor A) has the distance between them equal 40 mm and the other (Sensor B) 80 mm. Both sensors are made of the polyoxymethylene having apparent permittivity value equal 3.8. Waveguides are made of brass plate bars, 10 × 2 mm in dimension and 200 mm long, that are soldered to a two-line printed circuit which connects them to BNC con-nector for coaxial cable. Figure 2 presents the photographs of both sensors.

### 2.3. Microwave Measuring Setup

**Figure 3:**
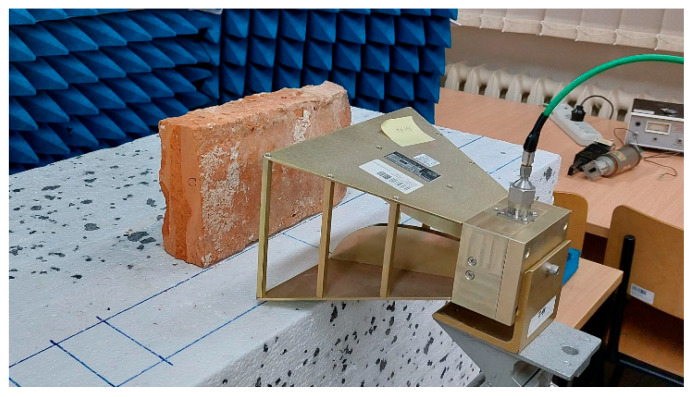
Close up of the laboratory setup during microwave measurements: double ridge antenna, a brick, styrofoam stand and microwave absorbers.

The microwave measuring setup used in this work consisted of a VNA model Agilent N5224A and a double-ridge pyramidal horn antenna. The antenna was placed at a distance of 8 cm from the sample surface. The surrounding area was covered by microwave absorbing materials in order to ensure reflection-free conditions. A picture of the measurement setup is shown in Figure 3.

### 2.4. Methods

#### 2.4.1. Reflectometric Investigation

Research has been conducted under constant temperature conditions 20 ± 1 °C and air relative humidity at the level of 50%. All samples in the range of moisture between dry 0%_mass_ and fully saturated material 14%_mass_ were examined by two types of TDR non-invasive sensors (Sensors A and B) described in Section 2.2 which differed in width. For statistical analysis, the measurements were repeated 5 times for each sensor and material sample.

#### 2.4.2. Microwave Investigation

Investigations of the prepared bricks with the use of microwave measuring method have been conducted according to the scheme presented in Figure 1 and the test-bed shown in Figure 3.

The VNA has been calibrated for the use of one port S_11_ measurements. The frequency range has been set as from 1 GHz to 11 GHz. The measurements results are in the form of complex values of reflection coefficient S_11_ (magnitude and angle), which are instantly recalculated by VNA to time domain. This allows visualizing the sounding wideband pulse reflected from inhomogeneities of the media along the wave propagation direction.

The main reflected peak is caused by the impedance difference between the air and the brick surface (the wave entering the brick material). The next significant peak is created by the reflection of the wave exiting the brick on the opposite side (the same boundary, material–air). The distance between these peaks in time domain carries the information of propagation time inside the brick material, the value is doubled due to round-trip propagation. As the brick thickness is known, the wave propagation velocity inside the brick material, and the real part of relative permittivity of the material, may be calculated.

The measurements of the prepared bricks have been performed for the whole brick set. For the purposes of statistical processing, all single brick measurements have been repeated 5 times. 

### 2.5. Data Analysis Method

The results achieved with the conducted research consist of the dependences between material mass moisture of the examined material sample and apparent permittivity readouts achieved by both TDR sensors and microwave antennas.

With these results, suitable regression models were applied and measurement uncertainty evaluation was conducted according to GUM guide [GUM] [69].

For calibration, polynomial dependence was applied according to the following formula [55]:

(4) $$\hat{w}=\beta_{0}+\beta_{1}\cdot \varepsilon +\beta_{2}\cdot \varepsilon^{2}+\epsilon$$

where $\;\hat{w}$ denotes mass moisture value estimated using polynomial model [%_mass_], *ε* the apparent permittivity measured using TDR, and *ϵ* the random error of normal distribution.

According to many articles [63,70,71] uncertainty was expressed as root mean squared error (RMSE), which allows expressing model uncertainty and quality of model fit to the measured data.

Uncertainty evaluation covers evaluation of combined uncertainty, being the combination of two types of uncertainties: A type, i.e., the statistical uncertainty that is dependent on fitting quality of the assumed model as well as uncertainty of B type, dependent on both uncertainties and resolutions of particular devices. With the combined standard uncertainty, the expanded measuring uncertainty was estimated.

For all applied measuring methods, the B type uncertainties are significantly lower than the A type uncertainty and neglected in the calculation, so the following elements are considered for uncertainty evaluation: *β*_0_, *β*_1_, *β*_2_ estimators, and the relative permittivity (*ε*):

(5) $$w=f\left(\beta_{0},\beta_{1},\beta_{2},\varepsilon \right)$$

The above-mentioned combined standard measurement uncertainty that includes both A and B type uncertainties can be described using the following formula [69,72]:

(6) $$u_{C}\left(w\right)=\sqrt{{\left(\frac{\partial w}{\partial \varepsilon }u\left(\varepsilon \right)\right)}^{2}+\overset{2}{\underset{i=0}{\sum }}{\left(\frac{\partial w}{\partial \beta_{i}}u\left(\beta_{i}\right)\right)}^{2}+2\overset{2}{\underset{i=0}{\sum }}\overset{2}{\underset{j=i+1}{\sum }}\frac{\partial w}{\partial \beta_{i}}\frac{\partial w}{\partial \beta_{j}}u\left(\beta_{i},\beta_{j}\right)}$$

so:

(7) $$u_{c}{}^{2}\left(w\right)=S^{2}\left(1+\frac{1}{n}+\sum_{i}{\left(\frac{\partial w}{\partial \beta_{i}}\right)}^{2}u^{2}\left(\beta_{i}\right)+2\sum_{ij}\left(\frac{\partial w}{\partial \beta_{i}}\frac{\partial w}{\partial \beta_{j}}\right)cov\left(\beta_{i}\beta_{j}\right)\right)$$

And the expanded uncertainty with the following formula:

(8) $$U\left(w\right)=k_{p}\cdot \;u_{c}\left(w\right)$$

where *k_p_* denotes the coverage factor that depends on the number of degrees of freedom and oscillates about 2 in value.

## 3. Results

Raw results achieved from both reflectometric multimeter and microwave analyzer are presented in the form of the waveforms that needed suitable interpretation to evaluate the relative permittivity value.

### 3.1. TDR Sensors Readouts

**Figure 4:**
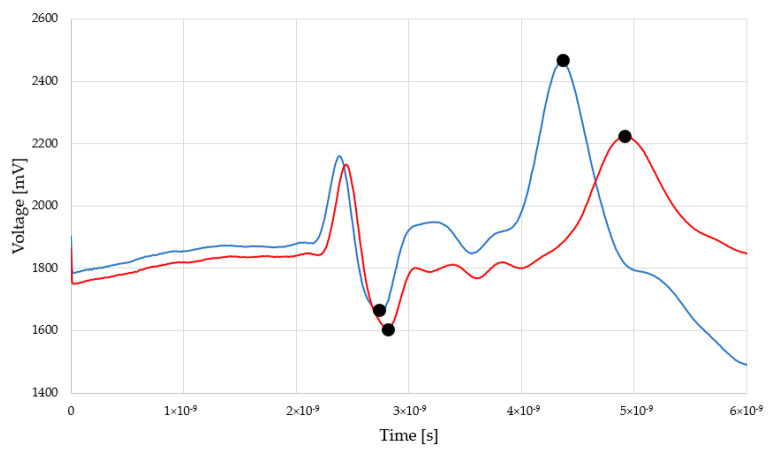
Reflectograms achieved by Sensor A for dry and moist material.

**Figure 5:**
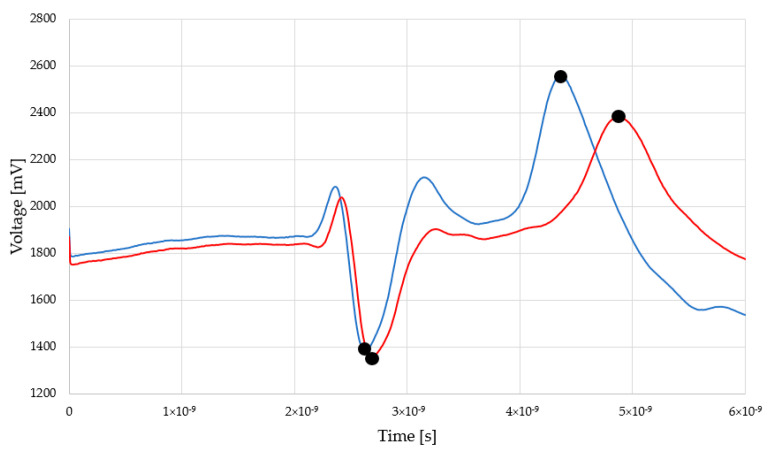
Reflectograms achieved by Sensor B for dry and moist material.

Exemplary reflectograms achieved by TDR Sensor A and Sensor B for dry and saturated status are presented in Figure 4 and Figure 5.

**Figure 6:**
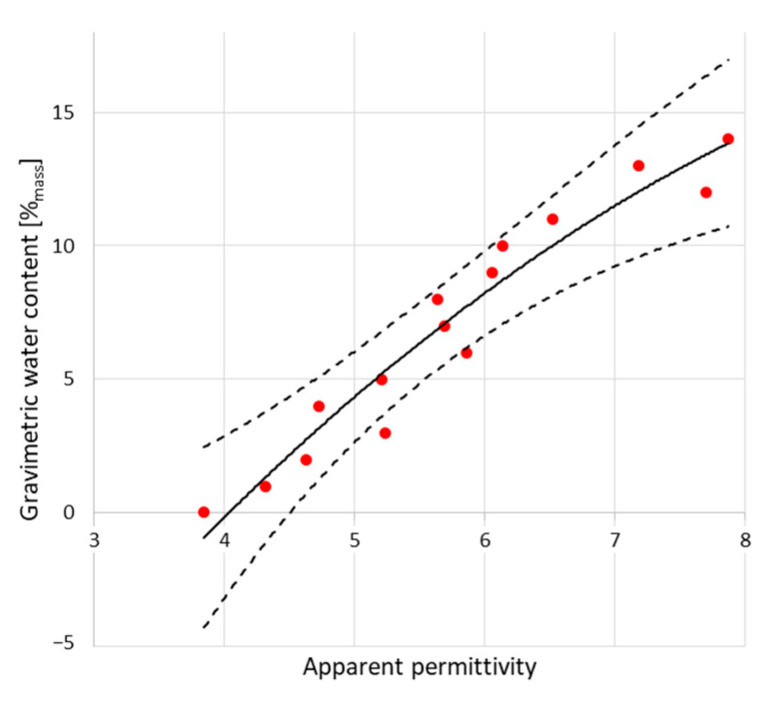
Dependence between relative permittivity readouts using TDR A Sensor and mass moisture evaluated gravimetrically.

**Figure 7:**
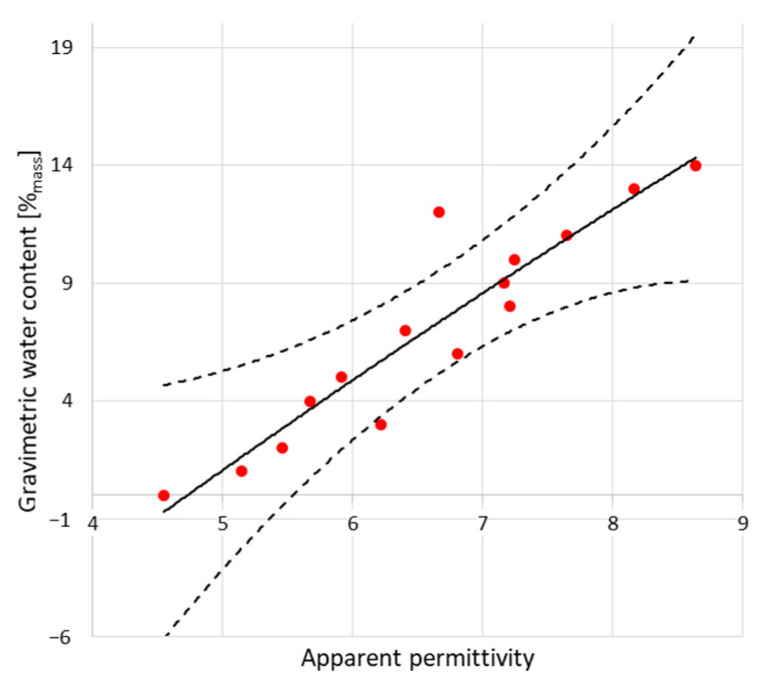
Dependence between relative permittivity readouts using TDR B Sensor and mass moisture evaluated gravimetrically.

The peaks indicated by black dots are markers for electromagnetic pulse travel along the waveguides touching the examined material. In both figures, the first, negative one shows the entrance to the sensor (built-in resistor) and the second (positive) one, sensor termination. The position of this second peak is significant for relative permittivity evaluation. In the case of dry material (blue line), the second position is located closer to the first, negative peak. In the case of moist material (red line), second peak is located further. Within the measurement, a set of the TDR waveforms was collected and the distances between markers were recalculated into the relative permittivity values according to the Equation (2). Figure 6 and Figure 7 present the recalculated times of signal propagation into relative permittivity values for different material humidity, showing the relations between red brick mass moisture and relative permittivity values, whereas dashed lines mean confidence intervals. Red dots represent the mean values of the five relative permittivity readouts for each material moisture content.

### 3.2. Microwave Readouts

**Figure 8:**
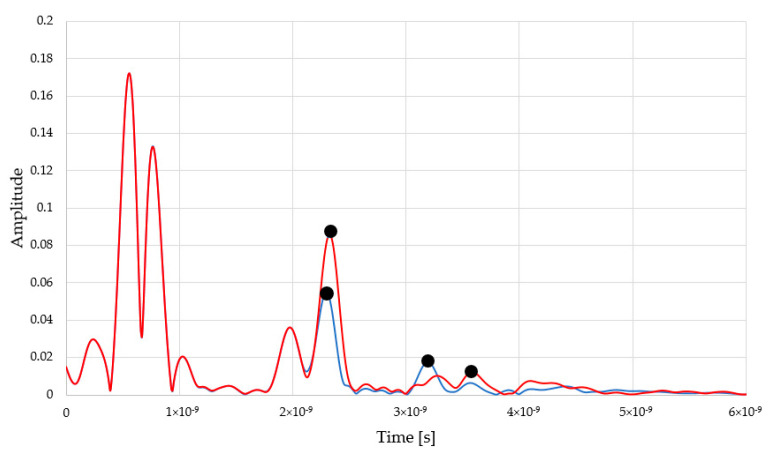
Exemplary waveforms achieved for 3%_mass_ and 9%_mass_ moist material with the use of microwave reflectance sensor.

Similarly to reflectometric readouts, the waveforms achieved from microwave measurement are presented in Figure 8. 

**Figure 9:**
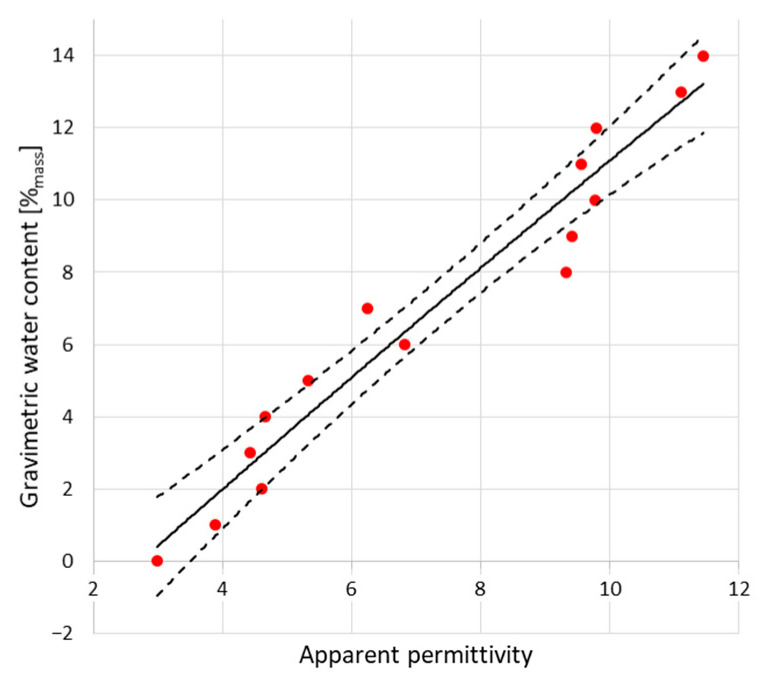
Dependence between relative permittivity readouts using microwave antenna and mass moisture evaluated gravimetrically.

The positive peaks highlighted by using black dots are the markers for electromagnetic pulse travel. This time, the first peak is the reflection from the first brick surface and the second peak represents the back of the material. The first peaks are located more or less in a similar position. In turn, the second peaks are shifted towards the right, depending on the moisture status of the material. In the case of moist material (9%_mass_), the second peak is shifted towards the right, compared to drier brick (3%_mass_). The two peaks below 1ns are associated with the pulse response of the antenna structure and do not influence the measurement of a distant object. Figure 9 presents the relation between red brick mass moisture and the relative permittivity values derived from measurements.

## 4. Discussion

With the data obtained using reflectometry and microwave measurements, some dependencies have been found and described with empirical second order polynomial regression formulas.

### 4.1. Regression Models

In the case of both TDR sensors and microwave antenna, together with the moisture increase, a relative permittivity increase is noticed. For dry samples, average permittivity readouts show the value of 4 in the case of TDR sensor A and 4.5 for TDR sensor B. For saturated states, the permittivity readouts are equal to 8 for sensor A and 8.5 for sensor B. In the case of the microwave antenna for dry material, the measured level of relative permittivity equaled about 3, while the readouts of moist material provided the relative permittivity values equal to 14. These differences could be attributed to the construction of the sensors. The relative permittivity read by the non-invasive sensors is an averaged value of the relative permittivity of the measured material, but also the sensor cover that is made of polyoxymethylene having relative permittivity constant in value, equal to 3.8 [35]. In the case of the microwave antenna sensor, the signal travels free space and totally penetrates the moist material and the second marker comes from the end of the brick, rather than the end of antenna, which provides more reliable readouts of brick moisture. Moreover, it must be emphasized that the range of TDR signal penetration for the applied sensors equals about 4–5 cm, which was evaluated in the following reference [56]. The values of relative permittivity measured for dry bricks could be compared to those found in the literature, both for microwave antenna and the TDR investigations. According to Pinhasi et al. [73], the value of dry red brick real permittivity equals 3.3 and the brick wall permittivity equals 3.56. Choroszucho et al. [74] stated that the value of dry brick permittivity equals 4.4, while Kaiser et al. [75] evaluated the permittivity of red brick at the level of 4.1. A comparison of relative permittivity of moist bricks is hindered by the different water absorptivity of materials. Nevertheless, it could be stated that this value differs between 15 and 20, depending on material type [76,77], and is higher than the one achieved in this investigation.

Table 2 presents the regression models achieved for all tested sensors (TDR Sensor A,B and microwave antenna) together with the coefficient of determination (R^2^), residual error (RSE), root mean square error (RMSE), and F statistics.

The assumed model of the relation between gravimetric water content and relative permittivity values based on second degree polynomial fit is satisfactory for both TDR and microwave sensors. The calibration models presented in Table 2 describe the relation between both measured values as of good quality, which is confirmed by high values of the coefficients of determination (R^2^) that exceed the value of 0.90 in two cases; only in the case of TDR sensor B, it equals 0.87. The best values were achieved by microwave antenna sensor (nearly 0.95), but TDR A sensor is not significantly worse (R^2^ of about 0.94). These high values of R^2^ mean that only about of 5% of the estimated value of gravimetric water content $\hat{w}$ is not properly described by the assumed model.

The values of RSE (residual standard error) vary between 1.12 and 1.76%_mass_. The smallest value is noticed for microwave antenna sensor, whereas the highest one is the TDR, wide sensor B. The RMSE (root mean square error) values, being a popular measure of the uncertainty, range here between 1.00 and 1.57%_mass_, depending on the sensor applied. The lowest and the best values are again noticed for the microwave antenna, and the highest for TDR Sensor B. Similar to the RSE coefficient, the RMSE values found in literature are mostly expressed in volumetric water content (%_vol_ or cm^3^/cm^3^) which hinders the comparison of these numbers. Nevertheless, it can be noticed that those values are comparable in value or sometimes lower than those found in the literature concerning electric moisture measurement techniques. The RMSE values achieved by Ju et al. [78] utilizing the Topp’s [34] dependence between relative permittivity and material moisture vary between 1 and 6.6%_vol_. In another research, presented by Roth et al. [70], the RMSE values equaled 0.8–3.7%_vol_, depending on material. The calibration method proposed by Malicki’s [63] enabled to achieve the RMSE values at the level of about 3%_vol_. Cubic polynomial model by Byun et al. [79] provided the RMSE values between 4 and 5%_vol_, depending on the material. Moreover, it should be emphasized that the RMSE values achieved by the tested TDR and microwave sensors are developed precisely for the examined material and particular sensor specimen. In the previously cited research, the applied models are universal and, in many cases, may not influence some characteristic data. When the achieved RMSE values are compared to those in the research presented by Udawatta et al. [64], with developed individual models for moisture evaluation by the invasive TDR probes, it can be noticed that RMSE varied in the range of 0.8–3.4%_vol_. In most cases, they were lower than the RMSE values established for the flat surface sensors. It is interesting that Domínguez-Niño et al. [80] conducted research in which they compared the RMSE values for the universal models, but also specific for particular sensors and materials, and achieved the RMSE values for the specific models at very low level (between 0.5 and 1%_vol_) as well as between 0.9 and 1.5%_vol_ for typical, universal models applied for moisture evaluation. Those values are comparable to the RMSE values achieved in the research presented in this article. At the end, it should be noticed that the RMSE values characterizing the methods and sensors presented in this research could be compared to the another research by the co-authors of this article (Suchorab et al.) [55] where the RMSE values ranged between 2.4 and 3.2%_vol_ for simplified sensors construction.

**Figure 10:**
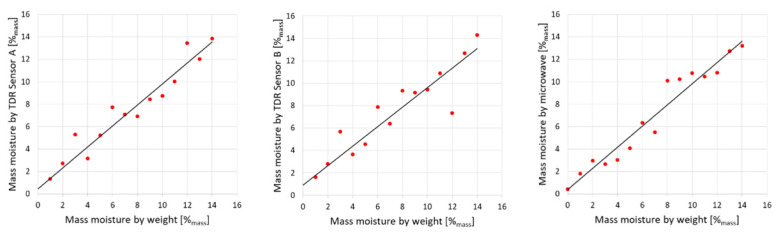
Dependences between material moisture evaluated gravimetrically and using various electric sensors. Red dots denote measuring points.

Figure 10 presents the results achieved from applied regression models evaluated for all sensors and the gravimetric evaluation. Linear dependences are presented in Table 3.

The dependence between the gravimetrically determined moisture (*w*) and TDR/microwave evaluated moisture value $\hat{w}$ is clearly linear, which is visible in the diagrams presented in Figure 10. In all cases, the slope values ranged between 0.868 (the worst, for TDR Sensor B) and 0.946 (microwave antenna sensor, the best). The slope values are smaller compared to other studies by the co-authors of this paper [36,55] that were conducted using TDR A sensor specimen, but for a different material (aerated concrete), obtaining 0.994, and other non-invasive sensors which achieved the slope values between 0.988–0.993.

### 4.2. Uncertainty Evaluation

**Figure 11:**
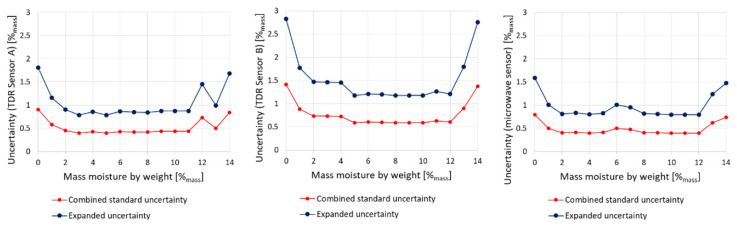
Distribution of combined standard and expanded measurement uncertainties for red brick and all tested sensors.

The detailed uncertainty analysis covers the evaluation of the combined standard uncertainty and complex uncertainty in relation to brick moisture. The results are presented graphically in Figure 11.

From the diagrams presented in Figure 11, it can be noticed that both types of uncertainty vary depending on the moisture status. The lowest values of uncertainty are observed for intermediate moisture states (between 2 and 12%_mass_). Under nearly dry or saturated conditions, those values are the highest. These variations are typical for most measuring devices and here they are mainly caused by the type of the applied regression models for calibration [36]. The lowest values have been noticed for both types of sensors. In intermediate states of moisture, expanded uncertainty amounts to about 0.8–1.0%_mass_, while for TDR Sensor B it ranges between 1.1–1.5%_mass_. In dry and wet states, expanded uncertainty is higher than 1.5%_mass_ for both microwave antenna and TDR Sensor A and even 2.8%_mass_ for TDR Sensor B, which is of lower quality. 

While comparing the achieved results of uncertainty evaluation, it should be emphasized that they are similar or better than those found in the literature, where they vary between 1 and 4%_vol_, depending on the material and sensing technique. According to Černý [58], TDR uncertainty for moisture evaluation of building materials equals 2.69%_vol_, but Ju et al. [78] evaluated the reflectometric method uncertainty at the level of 4.0%_vol_. In turn, according to Roth et al. [70], the uncertainty value is between 1.1 and 1.3%_vol_, whereas Malicki et al. [63] reported the values between 0.4–1.8%_vol_. Comparing to own previous research, it must be mentioned that the TDR Sensor A used in [36] reached the uncertainty level of 1.0%_vol_ for moderate values of moisture, while for dry and nearly saturated states, it was equal to even 2.0%_vol_. In the case of another construction of non-invasive TDR sensors presented in [55], the uncertainty values were higher and reached about 1.5%_vol_ in intermediate states and even more than 3%_vol_ for wet states.

The microwave measurements in this research have been performed with the use of broadband double-ridge horn antenna, which is a kind of “laboratory standard”. In order to implement this method in the real design, it seems necessary to develop a compact antenna with appropriate broad frequency bandwidth and high gain. For this purpose, it may be valuable to implement a kind of metamaterial technology, as shown in the exemplary references [81,82]. This approach would improve the performance of both microwave antennas and TDR sensors.

The values of the expanded uncertainty characterizing the microwave antenna as well as both TDR sensors are similar or lower comparing to other, invasive and non-invasive sensors. Nevertheless, it has to be mentioned that most of these devices perform according to universal calibration approach, while the measurements could be disturbed by unconsidered factors, such as material porosity or chemical composition of the solid phase. Such beneficial values of this parameter are mainly obtained by the individual calibration of each sensor that covers the influences of the construction differences and material characteristics.

## 5. Conclusions

The research on the microwave antenna and two non-invasive TDR sensors presented in this article proves that these techniques could be applied in non-invasive determination of moisture states of the porous building materials. To achieve the satisfactory level of applicability, all proposed sensors demand individual calibration for each sensor design as well as material type. This is mainly caused by the structure of porous building materials that may differ in porosity, absorptivity, or bulk density as well as the chemical compound of the solid phase. Contrary to the universal calibration models, individual calibration covers material differences and thus positively influences the value of measurement uncertainty. 

Among the tested sensors, the microwave antenna sensor is characterized by the most beneficial measuring parameters, including the RMSE value for calibration formula equal to 1.00%_mass_ and expanded measuring uncertainty in intermediate moisture states in the range of 0.8–1.0%_mass_. Non-invasive TDR Sensor A, presented previously in another research, has the RMSE value equal to 1.09%_mass_ and expanded uncertainty in intermediate moisture states comparable to the microwave antenna sensor. The worst measured parameters were achieved by TDR Sensor B with RMSE equal to 1.57%_mass_ and expanded uncertainty reaching 1.5%_mass_.

Nevertheless, it should be emphasized that all tested sensors provide satisfactory measuring features compared to other, even invasive sensors that are not individually calibrated for particular sensor construction or material.

All of the applied sensors are prospective for non-invasive masonry moisture evaluation, but it should be remembered that both TDR sensors are designed for surface testing and enable to detect condensation moisture in the surface layers of the masonry. In turn, microwave antenna enables penetration across the total thickness of the brick or even entire masonry thickness and enables detection of interior masonry moisture, even capillary water. 

## Figures and Tables

**Table 1:** Parameters of solid ceramic bricks adopted for testing.

Dimensions [cm]	Mass in Dry State [g]	Added Water [g]	Mass after Soaking [g]	Moisture Content by Mass [%]	Moisture Content by Volume [%]	Apparent Density [g/cm^3^]
25.2 × 11.9 × 5.9	3385.2	33.9	3419.1	1	1.9	1903.3
25.3 × 12.0 × 6.0	3332.4	66.6	3399.0	2	3.7	1833.0
25.2 × 12.0 × 6.1	3398.4	102.0	3500.4	3	5.5	1842.5
25.1 × 11.9 × 6.0	3396.2	135.8	3532.0	4	7.6	1888.3
25.4 × 12.0 × 6.1	3461	173.1	3634.1	5	9.3	1853.9
24.9 × 11.9 × 6.0	3290.4	197.4	3487.8	6	11.1	1848.9
25.3 × 11.9 × 6.2	3374.6	236.2	3610.8	7	12.8	1822.6
25.3 × 12.0 × 6.1	3270.2	261.6	3531.8	8	14.3	1787.1
25.2 × 12.0 × 5.9	3350	301.5	3651.5	9	16.9	1877.6
25.2 × 11.9 × 6.1	3329.6	333.0	3662.6	10	18.2	1817.6
25.3 × 12.0 × 6.1	3427	377.0	3804.0	11	20.6	1874.7
25.1 × 11.9 × 6.1	3318	398.2	3716.2	12	21.9	1821.1
25.3 × 12.0 × 6.0	3410.4	443.4	3853.8	13	24.2	1858.5
25.0 × 11.9 × 6.0	3313.6	463.9	3777.5	14	26.1	1860.9
25.2 × 11.8 × 5.8	3267.2	457.4	3724.6	14	26.6	1903.5

**Table 2:** Calibration models for TDR A and B sensors and microwave antenna.

Sensor	Regression Model	R^2^	RSE [%_mass_]	RMSE [%_mass_]	F Statistics
TDR—sensor A	*w* = −0.3088∙*ε*^2^ + 7.2882∙*ε* − 24.393	0.937	1.22	1.09	88.821 (***) (df = 2; 12)
TDR—sensor B	*w* = −0.067∙*ε*^2^ + 4.5585∙*ε* − 20.066	0.868	1.76	1.57	39.301 (***) (df = 2; 12)
Microwave antenna	*w* = −0.0074∙*ε*^2^ + 1.6193∙*ε* − 4.3662	0.946	1.12	1.00	105.463 (***) (df = 2; 12)

*** *p* < 0.001 (critical level of significance).

**Table 3:** Relations between gravimetric water content measured gravimetrically and evaluated by indirect techniques.

Sensor	Formula	R^2^
TDR—sensor A	y = 0.9368∙x + 0.4514	0.937
TDR—sensor B	y = 0.8676∙x + 0.9272	0.868
Microwave antenna	y = 0.9463∙x + 0.3767	0.946

## Data Availability

Data are contained within the article.
